# PMAT enhances sexual dimorphism of fear behaviors and facilitates female mice’s generalized contextual fear extinction

**DOI:** 10.3389/fnins.2026.1693593

**Published:** 2026-05-07

**Authors:** Aliyah J. Ross, Lauren R. Scrimshaw, Lauren N. Stoner, Jasmin N. Beaver, Grace A. Sonick, Lee M. Gilman

**Affiliations:** 1 Department of Psychological Sciences, Kent State University, Kent Campus, Kent, OH, United States; 2 Brain Health Research Institute, Kent State University, Kent Campus, Kent, OH, United States; 3 Healthy Communities Research Institute, Kent State University, Kent Campus, Kent, OH, United States

**Keywords:** anxiety disorder risk, learning and memory, monoamines, sex differences, transporters

## Abstract

Enhanced signaling of dopamine and/or serotonin during highly arousing situations can be reduced in part by monoamine transporters, such as plasma membrane monoamine transporter (PMAT, *Slc29a4*). An absence of selective pharmacological inhibitors means genetically modified mice constitutively deficient in PMAT remain the best tool for studying PMAT’s organism-level functional effects. Fear conditioning is a high arousal process. Generalization of fear is evolutionarily advantageous, whereby information learned from one experience is applied to other new but similar encounters. Pathological fear generalization, in contrast, is a core feature of most anxiety disorders. Given our previous findings indicating PMAT function reduces male mice’s context fear and enhances extinction of female mice’s cued fear, we hypothesized PMAT would similarly reduce generalization (i.e., enhance discrimination) of context and cued fear in male and female mice, respectively. Our context and cued fear conditioning experiments in adult PMAT wildtype (+/+) and heterozygous (+/−) male and female mice partially supported our hypotheses. We discovered PMAT facilitates extinction of contextually generalized fear, plus subsequent extinction of context-specific fear, selectively in females. Moreover, when specific fear cues or contexts were temporally presented before cues or contexts that were similar enough to make generalization possible, PMAT enhanced biological sex differences. Growing evidence reports common PMAT polymorphisms elicit measurable effects when PMAT function is reduced. Thus, we suspect future experiments may reveal positive associations between PMAT polymorphisms and risk for anxiety disorder symptoms, particularly in people assigned female at birth. Inclusion of these genetic variations in pharmacogenomic analyses may prove therapeutically beneficial.

## 1 Introduction

During stressful or other memorable events, monoamine (e.g., dopamine, serotonin) release is elevated (Tidey and Miczek, 1996; Morrow et al., 1999; Zanoveli et al., 2009; Yokoyama et al., 2005; Keeney et al., 2006; Finlay et al., 1995) and, in part, regulated by uptake transporters (Gainetdinov and Caron, 2003; Vaughan and Foster, 2013; Hensler et al., 2013) (see reviews Gasser, 2019; Daws, 2021). Higher affinity, lower capacity (A > C) monoamine transporters like dopamine transporter (DAT, *Slc6a3*) or serotonin transporter (SERT, *Slc6a4*) have relatively strong affinities [*K*_m_; 0.08–5.2 μM (Chang et al., 1996; Gu et al., 1994; Morón et al., 2003; Kilty et al., 1991; Ortore et al., 2020; Pacholczyk et al., 1991; Inazu et al., 2003; Barker et al., 1998; Giros et al., 1991)] for their substrate(s) and slower transport rates [0.001–0.300 nmol/min/mg protein (Chang et al., 1996; Gu et al., 1994; Morón et al., 2003)]. In contrast, higher capacity and lower affinity (C > A) monoamine transporters, like plasma membrane monoamine transporter (PMAT, *Slc29a4*), have weaker *K*_m_ values for dopamine and serotonin [80–2,100 μM (Duan and Wang, 2010; Wang et al., 2014; Shirasaka et al., 2017; Zhu et al., 2010; Amphoux et al., 2006; Busch et al., 1998)] and faster transport rates [0.42–18.2 nmol/min/mg protein (Duan and Wang, 2010; Wang et al., 2014; Shirasaka et al., 2017; Zhu et al., 2010; Engel et al., 2004)]. Given the abundance of legal and illicit drugs acting upon DAT and SERT, research has historically focused more upon such A > C transporters than upon C > A transporters. Indeed, C > A-selective drugs remain elusive (Miura et al., 2017; Angenoorth et al., 2021; Wu et al., 2025) (see reviews Maier et al., 2021; Koepsell, 2021), hindering understanding of their functional contributions. Nevertheless, increasing evidence indicates C > A transporters influence processes ranging from olfaction (Sardar et al., 2023); to pharmacokinetics (Shirasaka et al., 2016; Chen et al., 2015; Batara et al., 2023; Dawed et al., 2019; Christensen et al., 2011; Morales-Rivera et al., 2024; Moeez et al., 2021); to emotions (Wu et al., 2025; Beaver et al., 2022; Weber et al., 2023; Gilman et al., 2018).

Dopamine and serotonin signaling is enhanced during high arousal situations like emotional memory formation and recall (Tidey and Miczek, 1996; Morrow et al., 1999; Zanoveli et al., 2009; Yokoyama et al., 2005; Keeney et al., 2006; Wert-Carvajal et al., 2022) (see reviews LaLumiere, 2014; Coray and Quednow, 2022), when C > A transporters are hypothesized to physiologically contribute more because A > C transporters are likely saturated. Unlike most other C > A transporters, PMAT’s preferential affinity for dopamine (*K*_m_ = 201–466 mM) and serotonin (*K*_m_ = 82.9–283 mM) (Duan and Wang, 2010; Shirasaka et al., 2017; Engel et al., 2004) over other monoamines like norepinephrine (*K*_m_ = 1,078–2,606 mM) (Duan and Wang, 2010; Engel et al., 2004), combined with PMAT’s dopamine and serotonin transport rates ≥2.5–105x faster [possibly up to 3,700–14,200x faster; 0.750–22.4 nmol/min/mg protein (Duan and Wang, 2010; Shirasaka et al., 2017; Engel et al., 2004)] than DAT or SERT (Chang et al., 1996; Gu et al., 1994; Morón et al., 2003), make PMAT an auspicious, yet largely unrecognized, monoaminergic regulator.

Indeed, both rare and common human *SLC29A4* polymorphism studies are increasingly reporting associations between genetically attenuated PMAT activity and measurable effects in people (Wu et al., 2025; Dawed et al., 2019; Christensen et al., 2011; Moeez et al., 2021; Pérez-Gómez et al., 2023; Moon et al., 2018). For example, multiple *SLC29A4* single nucleotide polymorphisms are implicated in metformin absorption to an extent that drug adherence, response to metformin, and type II diabetes symptoms are all adversely affected (Dawed et al., 2019; Christensen et al., 2011; Moeez et al., 2021; Pérez-Gómez et al., 2023; Moon et al., 2018). Considering PMAT’s metformin affinity (*K*_m_ = 1,320 mM) (Zhou et al., 2007) is markedly weaker than its affinity for dopamine or serotonin, *SLC29A4* polymorphisms could plausibly influence brain monoamine signaling, particularly when elevated. A recent phenome-wide association analysis even flagged *SLC29A4* as a key “druggable gene” for mitigating postpartum depression risk (Wu et al., 2025).

In the absence of selective PMAT inhibitors (Miura et al., 2017; Angenoorth et al., 2021; Wu et al., 2025), mice with constitutively reduced or ablated PMAT function (Duan and Wang, 2013) are currently the best way to study PMAT–via subtraction–on an organismal level. Using these mice, we have found PMAT sex-selectively enhances (males) or reduces (females) active coping behaviors depending upon stressor type, and attenuates male context fear expression (Beaver et al., 2022; Weber et al., 2023; Gilman et al., 2018). PMAT function may also facilitate initial cued fear extinction learning in females (Weber et al., 2023). To continue assessing sex- and stress-specific effects of PMAT function, and thus how common *SLC29A4* polymorphisms might influence people’s mental health, we used PMAT-deficient mice to study fear generalization and discrimination here. Specifically, we intentionally used only wildtype (+/+) and heterozygous (+/−), and not knockout (−/−), mice, to both minimize potential confounds of lifelong compensatory processes, and to maximize the translatability of our findings to the many *SLC29A4* functional polymorphisms (Dawed et al., 2019; Christensen et al., 2011; Moeez et al., 2021; Pérez-Gómez et al., 2023; Moon et al., 2018).

Fear generalization is a natural, cross-species behavioral phenomenon (see reviews Jasnow et al., 2017; Bergstrom, 2020; Struyf et al., 2015; Asok et al., 2019) that reduces cognitive load by enabling one fear learning experience to be applied to multiple future situations. For instance, if someone were stung by a hornet, they might subsequently avoid other flying insects (e.g., houseflies, honeybees, etc.) to protect themselves. Sometimes, however, fear generalization becomes too broad, or otherwise reaches a pathological state; for example, if the same person begins fearing butterflies and songbirds. *Pathological* fear generalization is a shared, hallmark symptom of multiple anxiety disorders including specific phobias, social anxiety disorder, and generalized anxiety disorder (Asok et al., 2019; Cooper et al., 2022; Wong and Beckers, 2021; Fang et al., 2025; Dymond et al., 2013; Ahrens et al., 2016; Roesmann et al., 2022; Greenberg et al., 2013; Labuschagne et al., 2010; Reutter et al., 2025). In 2019, the World Health Organization reported over 300 million people globally were suffering from anxiety disorders (World Health Organization, 2023), making these some of the most prevalent mental health conditions worldwide.

Fear generalization is one end of a spectrum; at the other end is fear discrimination, when a distinction between two similar stimuli is learned [e.g., distinguishing between fire and tornado alarms (Struyf et al., 2015)]. Experiments in rodents have evaluated putative neurobiological mechanisms influencing fear generalization and fear discrimination in rodents through both context (Beaver et al., 2022; Beaver et al., 2023; Dulka et al., 2015; Asok et al., 2019) and cued (Dulka et al., 2015; Gilman et al., 2015; Toth et al., 2012) paradigms. Here, we leveraged a context paradigm to assess how much broader environmental encoding generalizes or remains discriminatory after 4 weeks in PMAT-deficient mice, with or without a return to the original conditioning context. In different mice, we implemented two distinct, discrete sounds, only one of which was a conditioning cue, to evaluate how PMAT function affects generalization and discrimination of explicit auditory cues.

Previous observations indicated PMAT deficiency elevates females’ active coping (Gilman et al., 2018) while suppressing males’ (Beaver et al., 2022) during distinct inescapable stressors. Further, procedures different from those used here indicated PMAT likely attenuates males’ context fear and enhances extinction of females’ cued fear following conditioning to a single auditory cue (Weber et al., 2023). Collectively, these sex-specific outcomes led to us hypothesizing that +/− males would exhibit enhanced context fear generalization, whereas +/− females would display augmented cued fear generalization. Put another way, we hypothesized typical (intact) PMAT function would constrain males’ context fear generalization while facilitating females’ fear discrimination of auditory cues (Weber et al., 2023). Our overarching goal is to understand PMAT’s roles in fear generalization and fear discrimination processes, and thereby guide future determinations of how *SLC29A4* polymorphic genotyping and/or PMAT molecular targeting could influence anxiety disorder etiology, symptom severity, and/or treatment responsiveness.

## 2 Method

Extended information on all materials and methods are available in the Supplementary material.

### 2.1 Animals

All experiments used adult (≥90 days old) male and female PMAT-deficient mice (Duan and Wang, 2013) on a C57BL/6J background bred in-house. Males’ experiments were always run before females’, to minimize potential confounds female pheromones upon male behavior (Baum and Keverne, 2002; Kavaliers et al., 2001; Musso et al., 2017). Kent State’s Institutional Animal Care and Use committee approved all experiments, and conditions adhered to the National Research Council’s Guide for the Care and Use of Laboratory Animals, 8th Ed. (National Research Council, 2011).

### 2.2 Contextual fear conditioning

**Figure 1:**
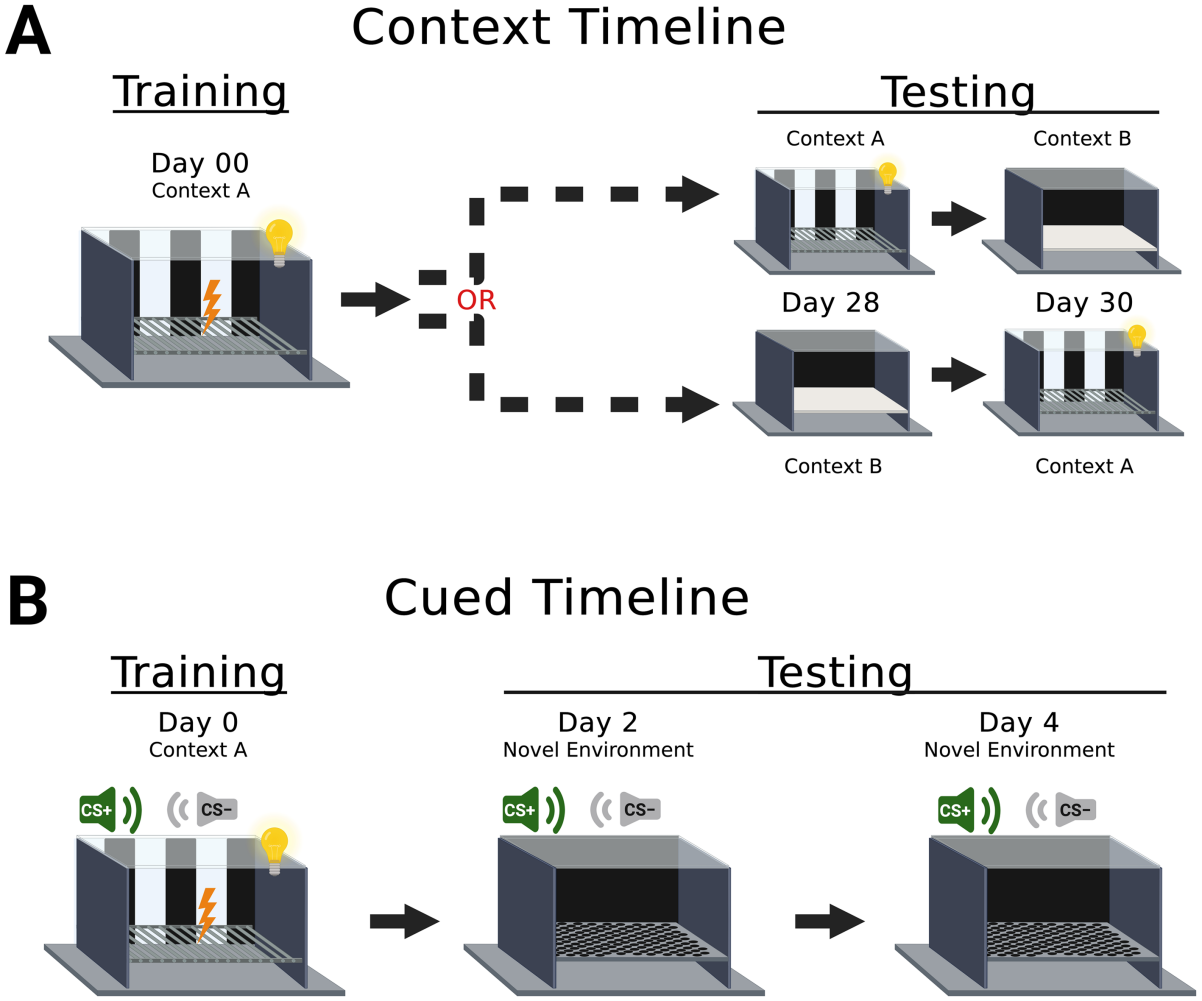
**Experimental timelines for context and cued fear**. Mice assigned to context fear were trained in context A on day 00 (panel **A**), then underwent testing on days 28 and 30. Context A consisted of a black and white striped background, shock grid floor, visible house light, and specific cleaning solution scent cue (70% ethanol). The sequence of testing was counterbalanced across mice, with some first tested in context A on day 28 then tested in context B on day 30, and others first tested in context B on day 28 then tested in context A on day 30. Context B consisted of a black background, smooth floor, infrared light, and a different cleaning solution scent cue (Windex®). Separate mice assigned to cued fear were trained in context A on day 0 (panel **B**). The conditioned stimulus (CS) that was explicitly paired with an aversive mild foot shock was an auditory tone at 7.5 kHz (CS+). The CS that was never paired with the aversive mild foot shock was an auditory tone at 2.0 kHz (CS−). On day 2, cued fear mice underwent cued expression testing and extinction training in a novel environment, which was the same as context B except the floor was stainless steel with circular holes. Testing of cued fear mice on day 4 in the novel environment was for cued fear extinction retention and additional extinction training. Aversive mild foot shocks were never administered during testing for context nor cued fear.

On day 0, mice underwent 6 minutes of context fear training (acquisition) (Weber et al., 2023; Beaver et al., 2023; Lynch et al., 2017; Cullen et al., 2015) in Context A (Figure 1A). Four weeks later, memory testing on day 28 (Beaver et al., 2023; Poulos et al., 2016; Wiltgen and Silva, 2007; Lotz et al., 2025) and day 30 lasted 10 min each. Context fear expression was tested in the training context (A), whereas context fear generalization was tested in the novel context (B). Testing in these two contexts was counterbalanced, meaning mice were either tested for fear expression at day 28, followed on day 30 by testing for fear generalization (A➜B); or mice were tested for fear generalization at day 28, followed on day 30 by fear expression testing (B➜A; Figure 1A). Freezing, i.e., absence of all movement except breathing, was quantified in real time using FreezeFrame 6 software (Actimetrics, Lafayette, IN).

### 2.3 Cued fear conditioning

Cued fear training (Weber et al., 2023; Gilman et al., 2015) occurred on day 0 in Context A (Figure 1B). Two auditory cues were utilized: one tone at 7.5 kHz (paired conditioned stimulus, CS+), and one tone at 2.0 kHz (unpaired conditioned stimulus, CS−). Mice experienced expression testing and extinction training on day 2, followed by extinction retention testing on day 4, both in a novel environment similar to Context B (Figure 1B).

### 2.4 Generalization and discrimination evaluations across fear paradigms

Discrimination and generalization are two ends of a spectrum (Bergstrom, 2020; Struyf et al., 2015), and consequently no universal thresholds exist at which one ends and the other begins. Rather, these processes are identified by utilizing previously established discriminable contexts or cues, and comparing how the procedurally optimized discrimination or generalization in the control group (here, +/+ mice) compares to that of the experimental manipulation group (here, +/− mice), as well as to how mice within each group respond to each of the contexts or cues they encountered during testing. For instance, testing context fear in Context B 28 days after training is an established timeline for producing context fear generalization in rodents (Beaver et al., 2023; Cullen et al., 2015; Poulos et al., 2016; Wiltgen and Silva, 2007; Lotz et al., 2025). If +/− mice displayed increased or decreased levels of fear in Context B on day 28 relative to +/+ mice, this would be one way to, respectively, detect enhanced context fear generalization or discrimination. Likewise, elevated or attenuated freezing of +/− mice during CS− testing, versus +/+ mice, respectively would suggest augmented cued fear generalization or discrimination (Gilman et al., 2015; Pollack et al., 2018).

### 2.5 Genotyping

Mice were weaned at postnatal day 21, at which time ear punches were taken for identification and genomic DNA extraction, followed by PCR then agarose gel electrophoresis (Beaver et al., 2022; Gilman et al., 2018; Duan and Wang, 2013).

### 2.6 Statistical analyses

Analyses were performed with IBM SPSS Statistics (v 31.0.0.0 (117), IBM, Armonk, NY, USA). The threshold for significance was set *a priori* at *p* < 0.05. Trends (*p* < 0.10) that did not reach this threshold were only acknowledged in text if the associated partial *η*^2^ (*η_p_*^2^) was ≥0.060. GraphPad Prism (v 10.6.1 (799), GraphPad Software, San Diego, CA, USA) was used to generate figures showing estimated marginal means (EM means) ± 95% confidence intervals (CIs) calculated in SPSS.

## 3 Results

None of our behavior measures produced a significant three-way interaction of time × genotype × sex.

### 3.1 Context fear

#### 3.1.1 Context fear training across both testing groups

**Figure 2:**
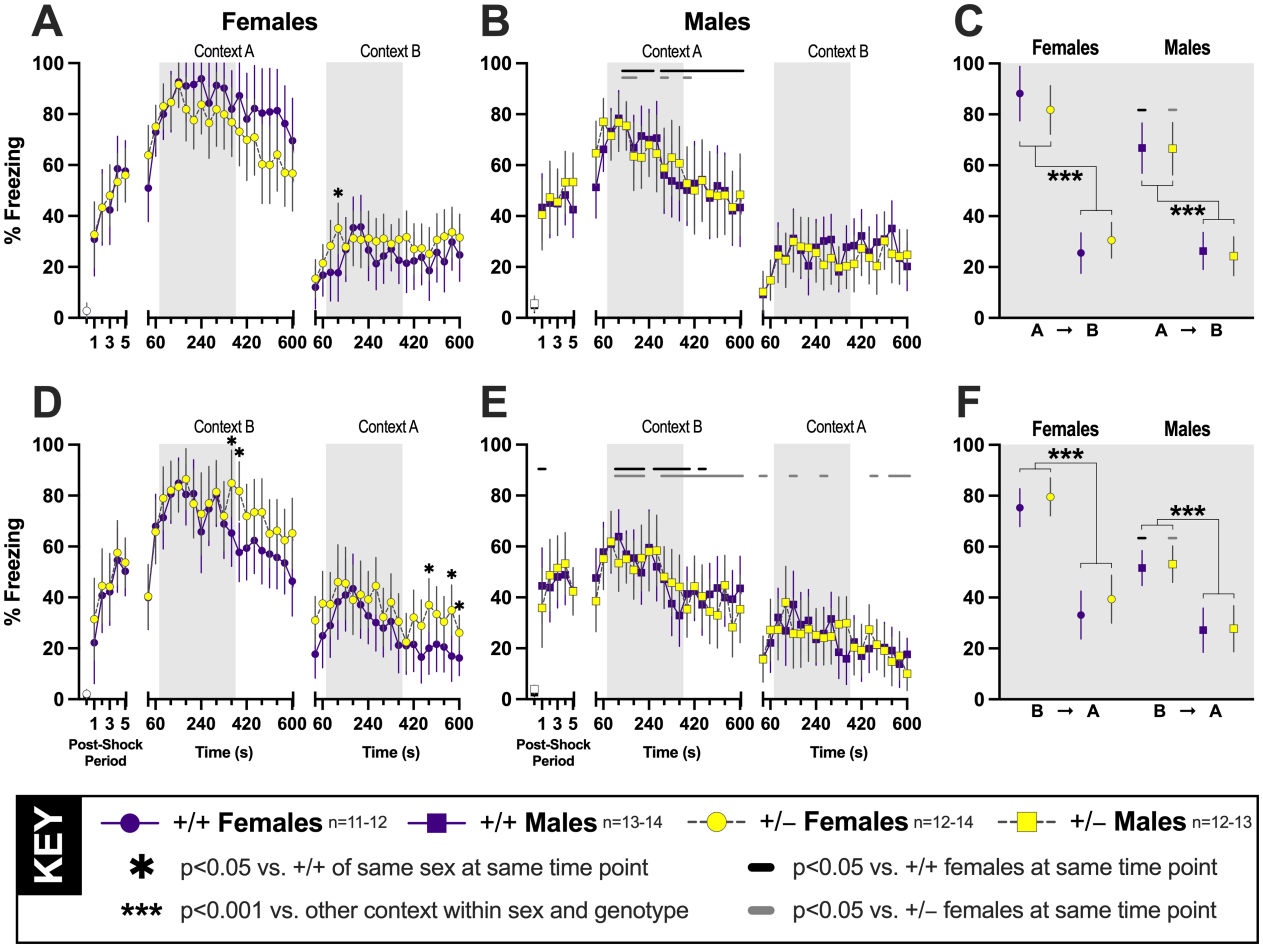
**Context fear expression and generalization were tested 4 weeks after contextual fear conditioning in PMAT-deficient mice**. Percent time spent freezing to the training context (Context A) or to a novel context (Context B) was measured in female (panels **A, D**; circles) and male (panels **B, E**; squares) mice. Solid purple symbols and lines indicate wildtype (+/+) data; yellow-filled grey symbols with dashed grey lines indicate heterozygous (+/−) data. All mice underwent context fear training (left third of each panel **A**, **B, D**, **E**), followed 28 days later (center third of each panel **A**, **B, D**, **E**) by testing in the training (panels **A**, **B**) or novel (panels **D**, **E**) context, then 48 h later (right third of each panel, **A**, **B, D**, **E**) the same mice were tested in the novel (panels **A**, **B**) or training (panels **D**, **E**) context. Averages of minutes two through six for each of the two testing days (light grey shaded rectangles, panels **A**, **B, D**, **E**) are presented in panels **C** and **F** by testing sequence (**C**: testing in A➜B; **F**: testing in B➜A). The first point in the first third of panels **A**, **B, D**, **E** represents average baseline (two min) freezing for female (circles; **A, D**) and male (squares; **B, E**) wildtype (black symbols) or heterozygous (white-filled grey symbols) prior to any foot shock administration. *p = 0.022 (panel **A**), 0.038, 0.005, 0.024, 0.013, 0.044 (panel **D**, left to right) vs. +/+ females within the same context testing sequence and time point. Horizontal black (+/+) or grey (+/−) bars indicate significant sex differences within genotype at the respective covered time point(s). For panels **(A)** vs. **(B)**, left to right, +/+ *p* = 0.013, 0.014, 0.024, 0.009, 0.003, 0.001, 0.005, <0.001, 0.042, 0.017, 0.007, 0.016, 0.006, 0.001, 0.025; +/− *p* = 0.016, 0.049, 0.035, 0.043. Panel **(C)**, +/+ *p* = 0.001, +/− *p* = 0.034 for context A males vs. females; ***p < 0.001 vs. other testing context within sex and genotype. For panels **(D)** vs. **(E)**, left to right, +/+ *p* = 0.046, 0.036, <0.001, 0.003, 0.001, 0.024, <0.001, 0.001, <0.001, 0.041, 0.006; +/− *p* = <0.001, <0.001, <0.001, 0.026, <0.001, 0.007, <0.001, <0.001, 0.002, <0.001, <0.001, 0.001, 0.015, <0.001, 0.003, 0.024, 0.049, 0.011, 0.034, 0.025, 0.012, 0.001. Panel **(F)**, +/+ *p* < 0.001 and +/− *p* < 0.001 for context B males vs. females; ***p < 0.001 vs. other testing context within sex and genotype. Data are graphed as EM means ± 95% CIs.

Context fear training of mice involved associating multiple previously neutral stimuli (olfactory, tactile, visual) with an aversive stimulus (mild foot shock). Context fear training did not reveal any genotype interactions, but significant time × sex interactions were observed both for mice intended to be tested first in the training context followed by the novel context, and for mice undergoing the reverse testing order (Tables 1, 2). No main genotype effects were found (Tables 1, 2). As observed previously (Weber et al., 2023), this indicates PMAT function does not have a detectable effect on the immediate context fear acquisition process. This suggests subsequent context fear behaviors during testing were not confounded by PMAT genotype influencing acquisition. Mice were intentionally counterbalanced after context training to further minimize risk of acquisition confounds affecting subsequent testing responses. Pairwise comparisons supported this outcome (Figure 2), with no genotype differences within sex at any time point, and only a single sex difference within wildtypes in the B➜A group for the first post-shock period during training (Figures 2D,E).

**Table 1:** Two-way RM GLMs of context training and testing responses for mice (to be) tested first in the training context (A), then in the novel context (B).

A➝B context mice			
Training	*F* statistic	*p*	*η_p_* ^2^
Time	*F*_(3.971,182.7)_ = 87.58	<0.001	0.656
Genotype	*F*_(1,46)_ = 0.101	0.753	0.002
Sex	*F*_(1,46)_ = 0.003	0.955	0.000
Time × Genotype	*F*_(3.971,182.7)_ = 0.266	0.898	0.006
**Time × Sex**	***F***_**(3.971,182.7)**_ **= 2.905**	**0.023**	**0.059**
Genotype × Sex	*F*_(1,46)_ = 0.082	0.775	0.002
Time × Genotype × Sex	*F*_(3.971,182.7)_ = 0.878	0.477	0.019
Testing Day 1	*F* statistic	*p*	*η_p_* ^2^
Time	*F*_(8.767,403.3)_ = 14.90	<0.001	0.245
Genotype	*F*_(1,46)_ = 0.524	0.473	0.011
Sex	*F*_(1,46)_ = 12.59	<0.001	0.215
Time × Genotype	*F*_(8.767,403.3)_ = 1.773	0.074	0.037
**Time × Sex**	***F***_**(8.767,403.3)**_ **= 3.178**	**0.001**	**0.065**
Genotype × Sex	*F*_(1,46)_ = 0.909	0.345	0.019
Time × Genotype × Sex	*F*_(8.767,403.3)_ = 0.730	0.677	0.016
Testing Day 2	*F* statistic	*p*	*η_p_* ^2^
**Time**	***F***_**(11.31,520.3)**_ **= 5.303**	**<0.001**	**0.103**
Genotype	*F*_(1,46)_ = 0.18	0.673	0.004
Sex	*F*_(1,46)_ = 0.285	0.596	0.006
Time × Genotype	*F*_(11.31,520.3)_ = 0.400	0.959	0.009
Time × Sex	*F*_(11.31,520.3)_ = 1.335	0.199	0.028
Genotype × Sex	*F*_(1,46)_ = 1.235	0.272	0.026
Time × Genotype × Sex	*F*_(11.31,520.254)_ = 1.301	0.218	0.028
Testing averages	*F* statistic	*p*	*η_p_* ^2^
Time	*F*_(1,46)_ = 421.9	<0.001	0.902
Genotype	*F*_(1,46)_ = 0.061	0.806	0.001
Sex	*F*_(1,46)_ = 8.031	0.007	0.149
Time × Genotype	*F*_(1,46)_ = 1.012	0.320	0.022
**Time × Sex**	***F***_**(1,46)**_ **= 10.59**	**0.002**	**0.187**
Genotype × Sex	*F*_(1,46)_ = 0.004	0.952	0.000
Time × Genotype × Sex	*F*_(1,46)_ = 1.914	0.173	0.040

Bold values indicate the highest level of interaction(s) and/or main effect(s) that reached significance upon analyses of the named data.

**Table 2:** Two-way RM GLMs of context training and testing for mice (to be) tested first in the novel context (B), then in the training context (A).

B➝A context mice			
Training	*F* statistic	*p*	*η_p_* ^2^
Time	*F*_(3.269,153.6)_ = 87.15	<0.001	0.650
Genotype	*F*_(1,47)_ = 0.229	0.635	0.005
Sex	*F*_(1,47)_ = 0.143	0.707	0.003
Time × Genotype	*F*_(3.269,153.632)_ = 0.158	0.936	0.003
**Time × Sex**	***F***_**(3.269,153.632)**_ **= 4.241**	**0.005**	**0.083**
Genotype × Sex	*F*_(1,47)_ = 0.089	0.767	0.002
Time × Genotype × Sex	*F*_(3.269,153.632)_ = 0.933	0.433	0.019
Testing Day 1	*F* statistic	*p*	*η_p_* ^2^
Time	*F*_(11.73,551.3)_ = 11.037	<0.001	0.190
Genotype	*F*_(1,47)_ = 0.73	0.397	0.015
Sex	*F*_(1,47)_ = 40.747	<0.001	0.464
Time × Genotype	*F*_(11.73,551.3)_ = 0.915	0.530	0.019
**Time × Sex**	***F***_**(11.73,551.3)**_ **= 3.258**	**<0.001**	**0.065**
Genotype × Sex	*F*_(1,47)_ = 1.602	0.212	0.033
Time × Genotype × Sex	*F*_(11.73,551.3)_ = 1.027	0.422	0.021
Testing Day 2	*F* statistic	*p*	*η_p_* ^2^
**Time**	***F***_**(9.014,423.7)**_ **= 8.007**	**<0.001**	**0.146**
Genotype	*F*_(1,47)_ = 1.636	0.207	0.034
**Sex**	***F***_**(1,47)**_ **= 4.299**	**0.044**	**0.084**
Time × Genotype	*F*_(9.014,423.7)_ = 1.322	0.223	0.027
Time × Sex	*F*_(9.014,423.7)_ = 0.949	0.483	0.020
Genotype × Sex	*F*_(1,47)_ = 1.346	0.252	0.028
Time × Genotype × Sex	*F*_(9.014,423.7)_ = 0.745	0.668	0.016
Testing Averages	*F* statistic	*p*	*η_p_* ^2^
Time	*F*_(1,47)_ = 202.8	<0.001	0.812
Genotype	*F*_(1,47)_ = 0.871	0.356	0.018
Sex	*F*_(1,47)_ = 25.11	<0.001	0.348
Time × Genotype	*F*_(1,47)_ = 0.013	0.910	0.000
**Time × Sex**	***F***_**(1,47)**_ **= 12.35**	**<0.001**	**0.208**
Genotype × Sex	*F*_(1,47)_ = 0.386	0.537	0.008
Time × Genotype × Sex	*F*_(1,47)_ = 0.100	0.753	0.002

Bold values indicate the highest level of interaction(s) and/or main effect(s) that reached significance upon analyses of the named data.

#### 3.1.2 Context fear testing on day 28 across both testing groups

To detect natural fear generalization that occurs over time, we tested PMAT +/+ and +/− mice in both groups 4 weeks after training. Day 28 testing for the A➜B group assessed context fear expression (Figures 2A,B), i.e., memory for the original training context, whereas day 28 testing for the B➜A group evaluated context fear generalization (Figures 2D,E). As with context training, context testing responses on day 28 did not result in any genotype interactions nor a main genotype effect, but the interaction of time × sex was again significant in both testing groups (Tables 1, 2). Initially, this could be interpreted as PMAT function having no effect upon context fear specificity nor generalization. However, careful assessment of pairwise comparisons indirectly revealed sex-specific effects of PMAT.

##### 3.1.2.1 Testing context-specific fear expression on day 28

Pairwise comparisons did not indicate any significant differences across genotype in either sex for mice tested first for context fear expression, then for context fear generalization (A➜B; Figures 2A,B). A sex difference was observed in +/+ mice, with pairwise comparisons highlighting that females exhibited greater freezing than males during 75% of day 28’s testing (Figures 2A,B). However, PMAT deficiency attenuated most of these sex differences on day 28, with only 20% of male +/− time points being significantly less than female +/− freezing (Figures 2A,B). This indicates that the full presence of PMAT typically augments females’, but not males’, specific context fear.

##### 3.1.2.2 Testing context fear generalization on day 28

In mice tested initially for context fear generalization, then for context fear expression (B➜A; Figures 2D,E), pairwise comparisons suggest a different pattern. Both genotypes across sexes exhibited fear generalization on day 28, and reduced PMAT function transiently prolonged this generalization in females, but not males (Figures 2D,E). This was made more apparent when comparing across sexes. While decreases in PMAT attenuated sex differences for A➜B mice, sex differences became more pronounced for B➜A +/− mice on day 28. Indeed, 75% of day 28 male +/− freezing time points were significantly less than +/− females, while only half of +/+ male timepoints were lower than +/+ females (Figures 2D,E). Thus, extinction of fear generalization may be enhanced by intact PMAT function in females, but not in males.

#### 3.1.3 Context fear testing on day 30 across both testing groups

Following day 28 testing for context-specific fear (A➜B) or context fear generalization (B➜A), day 30 tested for context fear discrimination as well as carryover effects of fear extinction from day 28. Deviating slightly from the preceding patterns, day 30 context testing responses for A➜B and B➜A mice displayed no two-way interactions (Tables 1-2). Both A➜B and B➜A day 30 behavior produced significant main effects of time (Tables 1-2), and a main effect of sex was also significant for B➜A mice (Table 2). Subsequent pairwise comparisons, in contrast to day 28, emphasized mild genotype effects selectively in B➜A mice.

##### 3.1.3.1 Testing context fear generalization on day 30, after context-specific fear expression testing

Unlike day 28, the predominant absence of significant pairwise comparisons for A➜B suggested no influence of PMAT on context fear generalization after a fear extinguishing testing session in the training context 2 days prior. A single time point was found by pairwise comparisons to indicate elevated freezing in +/+ as compared to +/− A➜B females on day 30 (Figure 2A), but otherwise no genotype nor sex differences were observed at any specific time points (Figures 2A,B).

##### 3.1.3.2 Testing context-specific fear expression on day 30, after testing context fear generalization

Akin to day 28 testing for B➜A mice, pairwise comparisons reported sustained elevations in B➜A +/− female freezing near the end of day 30 testing (Figure 2D), when extinction processes are typically more prominent. Moreover, female +/− mice in the B➜A testing sequence continued to exhibit elevated freezing during 35% of day 30’s time points vs. male +/− mice, whereas +/+ mice in this group displayed no significant pairwise comparisons across sex (Figures 2D,E). Thus, while less pronounced than day 28 testing, day 30 testing in B➜A mice indicates that intact PMAT function usually promotes females’ extinction of context-specific fear following a generalized fear experience.

#### 3.1.4 Average context fear testing across both testing groups and days

To focus on initial context fear expression or generalization, and evaluate discrimination between contexts in both possible sequences, while minimizing evaluation of extinction, we averaged freezing for all mice during minutes two through six on each of the two testing days (Weber et al., 2023; Beaver et al., 2023; Lynch et al., 2017). Analyses of these testing data reported significant time × sex interactions, without any genotype interactions or main effects (Tables 1, 2). Pairwise comparisons upheld these sex differences within genotypes, as well as the anticipated carryover effect of day 28’s fear extinction onto day 30 (Figures 2C,F). A non-significant trend (*p* = 0.080, *η_p_*^2^ = 0.064) was noted between female and male +/− mice in the B➜A group on day 30 (Figure 2F), aligning with our aforementioned observations. Moreover, discrimination between contexts regardless of testing sequence occurred for all sex and genotype combinations (Figures 2C,F), though admittedly this is inextricably associated with day 28 extinction processes.

### 3.2 Cued fear

**Figure 3:**
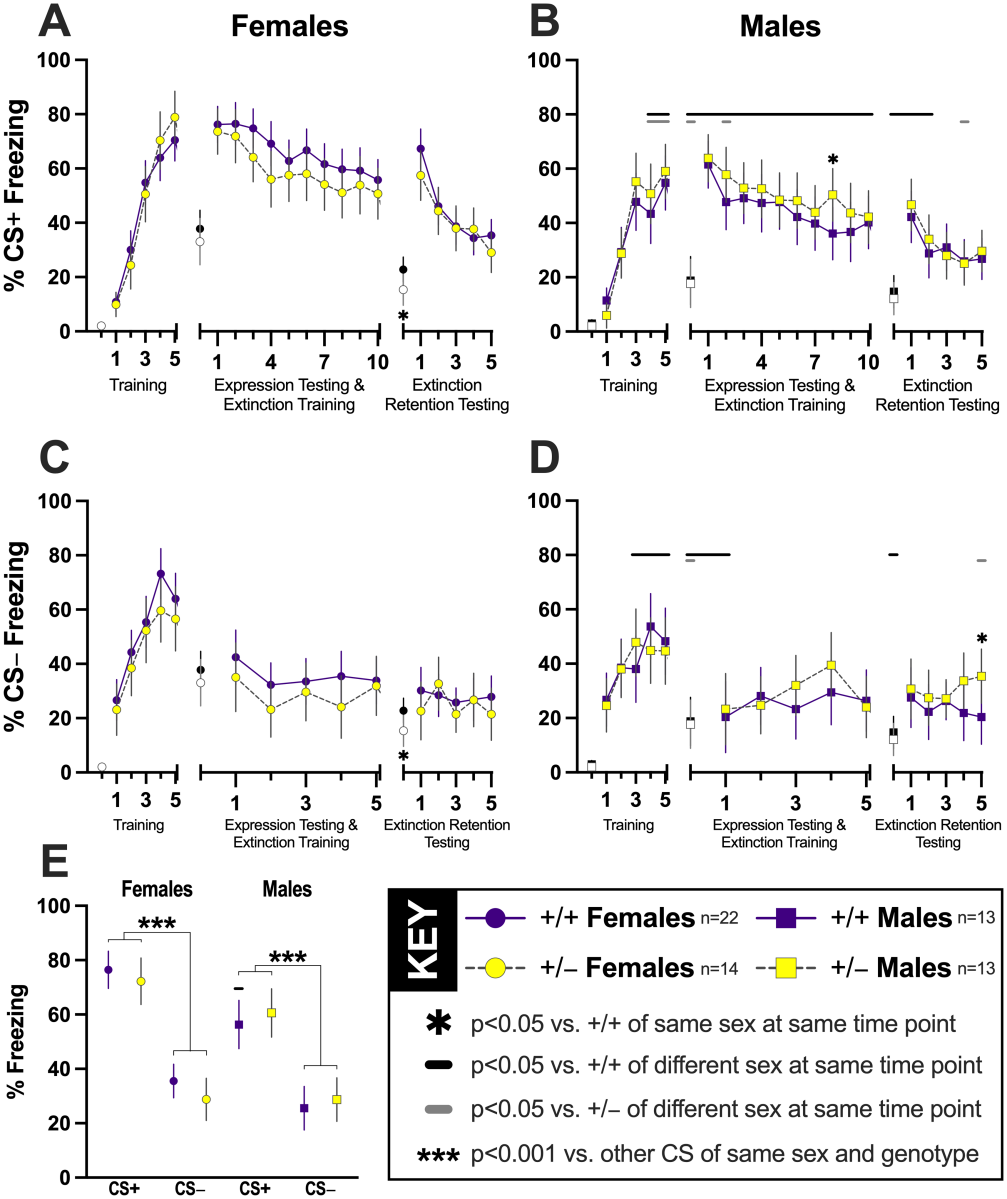
**Discrimination of explicitly paired and unpaired auditory cues during fear conditioning in PMAT-deficient mice**. Percent time spent freezing to the conditioned stimulus (CS) that was explicitly paired (CS+, 7.5 kHz; panels **A**, **B**) or never paired (CS−, 2.0 kHz; panels **C**, **D**) with an aversive mild foot shock are shown for female (panels **A, C**; circles) and male (panels **B, D**; squares) mice. Solid purple symbols and lines indicate wildtype (+/+) data; yellow-filled grey symbols with dashed grey lines indicate heterozygous (+/−) data. All mice underwent cued fear training (left third of each panel **A**–**D**) that employed five presentations each of the CS+ and CS−, followed 48 h later by cued expression testing and extinction training (center third of each panel **A**–**D**) involving 30 CS+ and 5 CS− tone presentations, and concluding 48 h thereafter by cued extinction retention testing (right third of each panel **A**–**D**) employing 15 CS+ and 5 CS− presentations. Freezing to CS+ on the two testing days are graphed as averages of three sequential CS+ presentations, creating 10 data points for the first testing day, and 5 for the second testing day. The first point in each third of panels **A**–**D** represents average baseline (two min) freezing for female (circles; **A, C**) and male (squares; **B, D**) wildtype (black symbols) or heterozygous (white-filled grey symbols) in the absence of any tones; thus, within sex and genotype, these baseline values are identical in panels **(A)** vs. **(C)** and **(B)** vs. **(D)**. Panel **(E)** shows the average percent time freezing during the first 5 CS+ and 5 CS− tones presented during cued expression testing and extinction training. *p = 0.046, 0.041, 0.046 vs. +/+ of same sex within CS and time point, in alphabetical panel order. Horizontal black (+/+) or grey (+/−) bars indicate significant sex differences within genotype at the respective covered time point(s). For panels **(A)** vs. **(B)**, left to right, +/+ *p* = 0.004, 0.015, 0.001, 0.009, <0.001, <0.001, 0.002, 0.020, <0.001, <0.001, <0.001, 0.002, 0.014, 0.034, <0.001, 0.003; +/− *p* = 0.012, 0.006, 0.016, 0.049, 0.026. For panels **(C)** vs. **(D)**, left to right, +/+ *p* = 0.028, 0.013, 0.047, 0.001, 0.010, 0.034; +/− *p* = 0.016, 0.049. Panel **(E)**, +/+ *p* < 0.001 for male vs. female CS+; ***p < 0.001 vs. other CS within sex and genotype. Data are graphed as EM means ± 95% CIs.

Distinct from contextual fear conditioning, where mice were counterbalanced after training across one of two testing sequences, mice that underwent cued fear conditioning here were all within the same group. Instead, all mice were presented with both CS+ and CS− auditory cues throughout training and testing; it is simply the responses to each of these that are graphed separately for clarity (Figure 3). Cued fear conditioning allowed us to examine PMAT’s influence upon encoding of, and fear responses to, two stimuli of the same modality. Only one was ever explicitly paired with the aversive foot shock, and this consequently permitted repeated assessment of cued fear discrimination or generalization in the same mice over a short (5 day) period.

#### 3.2.1 Cued fear training to CS+

Similar to context fear, analyses revealed a significant interaction between time × sex for CS+ responses during cued fear training (Table 3). Genotype was not a component of any significant two-way interactions nor a main effect (Table 3). As before, the time × sex interaction was expected, with females of both genotypes conditioning to the CS+ with higher freezing percentages than males, supported by pairwise comparisons (Figures 3A,B). Consequently, subsequent CS+ testing was unlikely to have been confounded by any genotype effects during acquisition.

**Table 3:** Two-way RM GLMs of cued training and testing responses to the CS explicitly paired with the aversive stimulus (CS+), and of cued ‘training’ and testing responses to the CS never paired with the aversive stimulus (CS−).

Cued CS+ responses			
Training	*F* statistic	*p*	*η_p_* ^2^
Time	*F*_**(4,232)**_ = 225.3	<0.001	0.795
Genotype	*F*_(1,58)_ = 0.322	0.573	0.006
Sex	*F*_(1,58)_ = 7.295	0.009	0.112
Time × Genotype	*F*_(4,232)_ = 1.593	0.177	0.027
**Time × Sex**	***F***_**(4,232)**_ **= 7.746**	**<0.001**	**0.118**
Genotype × Sex	*F*_(1,58)_ = 0.079	0.779	0.001
Time × Genotype × Sex	*F*_(4,232)_ = 0.765	0.549	0.013
Expression testing and extinction training	*F* statistic	*p*	*η_p_* ^2^
**Time**	***F***_**(6.673,387)**_ **= 59.71**	**<0.001**	**0.507**
Genotype	*F*_(1,58)_ = 0.068	0.795	0.001
**Sex**	***F***_**(1,58)**_ **= 16.51**	**<0.001**	**0.222**
Time × Genotype	*F*_(6.673,387)_ = 0.67	0.690	0.011
Time × Sex	*F*_(6.673,387)_ = 1.305	0.249	0.022
Genotype × Sex	*F*_(1,58)_ = 2.495	0.120	0.041
Time × Genotype × Sex	*F*_(6.673,387)_ = 1.125	0.346	0.019
Extinction retention testing	*F* statistic	*p*	*η_p_* ^2^
Time	*F*_**(4.347,252.1)**_ = 107.5	<0.001	0.650
Genotype	*F*_(1,58)_ = 0.189	0.666	0.003
Sex	*F*_(1,58)_ = 10.17	0.002	0.149
Time × Genotype	*F*_(4.347,252.1)_ = 1.179	0.321	0.020
**Time × Sex**	***F***_**(4.347,252.1)**_ **= 4.900**	**<0.001**	**0.078**
Genotype × Sex	*F*_(1,58)_ = 0.574	0.452	0.010
Time × Genotype × Sex	*F*_(4.347,252.1)_ = 2.230	0.061	0.037

Cued CS− responses			
Training	*F* statistic	*p*	*η_p_* ^2^
Time	*F*_**(4.071,236.1)**_ = 87.38	<0.001	0.601
Genotype	*F*_(1,58)_ = 1.493	0.227	0.025
Sex	*F*_(1,58)_ = 7.271	0.009	0.111
Time × Genotype	*F*_(4.071,236.1)_ = 1.211	0.307	0.020
**Time × Sex**	***F***_**(4.071,236.1)**_ **= 2.966**	**0.020**	**0.049**
Genotype × Sex	*F*_(1,58)_ = 0.709	0.403	0.012
Time × Genotype × Sex	*F*_(4.071,236.146)_ = 0.280	0.894	0.005
Expression testing and extinction training	*F* statistic	*p*	*η_p_* ^2^
Time	*F*_(4.591,266.3)_ = 0.872	0.493	0.015
Genotype	*F*_(1,58)_ = 0.316	0.576	0.005
Sex	*F*_(1,58)_ = 4.103	0.047	0.066
Time × Genotype	*F*_(4.591,266.3)_ = 0.437	0.807	0.007
**Time × Sex**	***F***_**(4.591,266.3)**_ **= 4.111**	**0.002**	**0.066**
Genotype × Sex	*F*_(1,58)_ = 1.623	0.208	0.027
Time × Genotype × Sex	*F*_(4.591,266.3)_ = 0.794	0.545	0.014
Extinction retention testing	*F* statistic	*p*	*η_p_* ^2^
**Time**	***F***_**(4.449,258)**_ **= 5.927**	**<0.001**	**0.093**
Genotype	*F*_(1,58)_ = 0.130	0.720	0.002
Sex	*F*_(1,58)_ = 0.007	0.931	0.000
Time × Genotype	*F*_(4.449,258)_ = 1.537	0.186	0.026
Time × Sex	*F*_(4.449,258)_ = 1.383	0.236	0.023
Genotype × Sex	*F*_(1,58)_ = 2.810	0.099	0.046
Time × Genotype × Sex	*F*_(4.449,258)_ = 0.983	0.423	0.017

Bold values indicate the highest level of interaction(s) and/or main effect(s) that reached significance upon analyses of the named data.

#### 3.2.2 Testing cue-specific (CS+) fear expression and extinction training

Two days after training, testing for CS+ cued fear expression concurrent with CS+ extinction training assessed how PMAT function impacted discriminative cued fear memory and extinction. Analyses resulted in no two-way interactions, but significant main effects of both sex and time were found (Table 3). These again support the anticipated greater freezing levels in females vs. males, along with time-dependent reductions in freezing across sexes, indicative of extinction (Table 3; Figures 3A,B). Pairwise comparisons of each genotype within sex indicated a single time point where +/− males displayed more freezing than +/+ males (Figure 3B). More informative are the pairwise comparisons across sexes at each genotype’s timepoint. Similar to context A➜B day 28 testing, +/+ males on the first day of cued testing displayed less freezing than +/+ females at all analyzed CS+ measures, whereas +/− males exhibited lower CS+ fear compared to +/− females for a mere 18% of that day’s measures (Figures 3A,B). In other words, typical PMAT function may enhance sex differences in cued fear discrimination of the CS+, given such sex differences are almost abolished in +/− mice.

#### 3.2.3 Testing cue-specific (CS+) fear extinction retention

Two days after the first testing session, mice were once more evaluated for their discrimination of the two cues, this time while retention of extinction was assessed. As with cued CS+ training, a significant interaction of time × sex was found without any genotype interactions nor a main genotype effect (Table 3). Pairwise comparisons indicated that +/− females had less baseline (i.e., prior to any cue presentations) freezing at the start of this extinction retention test than +/+ females (Figures 3A,C), the sole instance in this study where PMAT deficiency statistically attenuated fear behavior. The prior pattern of sex differences being more prominent between +/+ as opposed to +/− mice only partially (50%) persisted on this last cued testing day, disappearing for the last three CS+ measures for +/+ mice (Figures 3A,B). A single CS+ measure in +/− mice occurred where females froze more than males (Figures 3A,B). Akin to the context experiments, the influence of PMAT function on cued fear sex differences appears to largely diminish upon the second testing day.

#### 3.2.4 Cued fear ‘training’ to CS−

Considering CS− cues were interspersed between presentations of CS+ cues during every stage of cued fear training and testing, mice continually were required to discriminate between the two. For example, training involved explicit pairings of the mild foot shock with the CS+. Amidst these explicit pairings, presentations of the CS− occurred, but never was a foot shock paired with a CS−. For this cued CS− ‘training’, though no two-way interactions with, nor main effect of, genotype were observed, the interaction of time × sex was once more significant (Table 3). Pairwise comparisons found that for the last three measures of freezing to the CS−, +/+ but not +/− mice displayed sex differences, with +/+ males freezing less than +/+ females (Figures 3C,D). While some fear behavior is typical in response to the CS− (Gilman et al., 2015), despite it never being paired with a foot shock, our findings indicate that intact PMAT function could augment cued fear generalization during females’ acquisition.

#### 3.2.5 Testing cued fear generalization (CS−) and extinction training

Because of the genotype-specific sex difference noted for CS− ‘training’, the possibility of confounds during CS− testing existed. Indeed, the absence of a sex difference in +/−, but not +/+, mice persisted through the first CS− presentation on the cued expression testing and extinction training day as revealed by pairwise comparisons (Figures 3C,D). Also, as with the CS− ‘training’ day, the only significant two-way interaction was that of time × sex, and the main effect of genotype was not significant (Table 3). Therefore, it may be that the ‘training’ confound obscured what might have otherwise been a persistent elevation in +/− mice’s generalized fear to CS−; or it could be that the effects of this confound dissipated after the first CS− presentation.

#### 3.2.6 Testing cued fear generalization (CS−) extinction retention

Reflecting the majority of the initial CS− testing day, extinction retention testing for the CS− also revealed minimal genotype-specific sex differences. No two-way interactions emerged as significant, and the only main effect was that of time (Table 3). Pairwise comparisons indicated an unanticipated elevation in freezing during the final CS− presentation in +/− males as compared to +/+ males (Figure 3D), possibly indicating an emerging sensitization to the CS−. This time point is also the single instance here where male +/− mice exhibited heightened freezing behavior vs. +/− females. Otherwise, the only other difference was in baseline (i.e., cue-independent) freezing as mentioned previously, where +/+ females displayed more fear behavior than +/+ males.

#### 3.2.7 Average cued fear discrimination/generalization testing with first five CS+ and five CS− tones

Related to our focus on minutes two through six of context fear testing to more selectively assess fear expression while minimizing extinction processes, for cued fear we averaged the first five CS+ and five CS− tones played during the initial cued fear testing day. This facilitated clearer analyses of cued fear discrimination versus generalization, minimizing extinction influences, while still permitting sex and genotype analyses. Mirroring the patterns noted previously, time × sex was the only significant interaction, and no main effect of genotype was found (Table 4). Pairwise comparisons likewise illustrated clear sex differences in CS+ freezing responses across +/+ mice (Figure 3E), and further indicated a non-significant trend (*p* = 0.053, *η_p_*^2^ = 0.063) across sexes for CS− responses in +/+ mice. Notably, no sex difference was detected for CS+ responses in +/− mice (Figure 3E). Nevertheless, regardless of sex or genotype combination, all mice significantly discriminated between CS+ and CS− tones during expression testing. Thus, intact PMAT function does not appear to contribute to fear discrimination nor generalization processes as we hypothesized, but instead to canonical sexual dimorphism in freezing responses across context and cued fear conditioning paradigms.

**Table 4:** Two-way RM GLMs of the average responses to the first five CS+ and five CS− tones played during cued expression testing and extinction training.

First five CS+ and CS− cued fear expression	*F* statistic	*p*	*η_p_* ^2^
Time	*F*_**(1,58)**_ = 219.9	<0.001	0.791
Genotype	*F*_(1,58)_ = 0.078	0.782	0.001
Sex	*F*_(1,58)_ = 11.42	0.001	0.164
Time × Genotype	*F*_(1,58)_ = 0.138	0.712	0.002
**Time × Sex**	***F***_**(1,58)**_ **= 4.784**	**0.033**	**0.076**
Genotype × Sex	*F*_(1,58)_ = 2.212	0.142	0.037
Time × Genotype × Sex	*F*_(1,58)_ = 0.020	0.887	0.000

Bold values indicate the highest level of interaction(s) and/or main effect(s) that reached significance upon analyses of the named data.

## 4 Discussion

Contrary to our hypothesis, we discovered evidence indicating PMAT contributes to extinction of context fear generalization selectively in +/− females. Moreover, +/− females’ generalization experience appeared to subsequently impede context-specific fear extinction. Critically, these effects were not observed in males undergoing the same testing sequence, nor were they found when mice instead were first tested in the original training context prior to generalization testing. Combined, our data suggest reduced PMAT function in biological females impedes contextual generalization extinction and disrupts ensuing contextual extinction processes when the initial, specific contextual fear memory is retrieved. Whether this is through shared or distinct mechanisms requires additional investigation, but it aligns with previous work implicating PMAT in females’ extinction of cued fear (Weber et al., 2023).

To further investigate generalization and discrimination in PMAT deficient mice here, we used a two tone cued fear conditioning paradigm (Gilman et al., 2015). While we expected female-selective effects (Weber et al., 2023; Gilman et al., 2018), instead, PMAT reductions seemed to consistently mitigate sex differences observed between +/+ and +/− mice. A similar pattern was noted when testing A➜B mice in the training context. Thus, PMAT’s augmentation of these sexual dimorphisms appears to occur when first the *specific* context or cue is presented, followed thereafter by a context or cue that can elicit generalization. While our observations of PMAT’s effects here may be modest, consistencies within and across studies suggest PMAT could influence anxiety disorder risk, particularly in people assigned female at birth with at least one functional *SLC29A4* polymorphism (Dawed et al., 2019; Christensen et al., 2011; Moeez et al., 2021; Pérez-Gómez et al., 2023; Moon et al., 2018). Future work is needed to investigate if sex hormones organizationally contribute to PMAT’s interactions with dopamine and serotonin signaling, particularly regarding fear conditioning and extinction processes (Foilb and Christianson, 2016; Stubbendorff et al., 2023; Alves et al., 2022; Rey et al., 2014).

Limitations include use of constitutively deficient PMAT mice, meaning some lifelong compensatory processes may still have been engaged. Further, the magnitude of functional PMAT reduction in +/− mice remains elusive, due primarily to the absence of requisite molecular tools (Miura et al., 2017; Angenoorth et al., 2021; Wu et al., 2025; Yates et al., 2019; Dahlin et al., 2007). We intentionally did not monitor estrous cycles to observe outcomes in naturally cycling females, given evidence of irrelevance in mice (Asok et al., 2019; Meziane et al., 2007; Keiser et al., 2017) [but see rat cued fear findings (Milad et al., 2009; Trask et al., 2020)]. Because our goal was to optimize translational relevance and not to assess the necessity of PMAT, we did not study PMAT −/− mice here. Given PMAT’s functional contributions are most prominent under conditions of elevated monoaminergic signaling, we do not anticipate PMAT function is required; but only studies with PMAT −/− mice could assess such.

Our findings partially supported our hypotheses, plus implicated PMAT function [and potentially by extension, *SLC29A4* polymorphisms (Wu et al., 2025; Dawed et al., 2019; Christensen et al., 2011; Moeez et al., 2021; Pérez-Gómez et al., 2023; Moon et al., 2018)] in translationally relevant fear conditioning and extinction processes. Our data indicate intact PMAT function promotes females’, but not males’, extinction of both generalized and subsequent context-specific fear. Further, functional PMAT may contribute to sex differences in memory of unpleasant environments (Beaver et al., 2023; Asok et al., 2019; Keiser et al., 2017; Trott et al., 2022; Colon et al., 2018) and sounds temporally proximal to aversive experiences, even if unrelated (Gilman et al., 2015; Greiner et al., 2019; Day et al., 2016; Toufexis et al., 2007; Clark et al., 2019). In people, genetically attenuated PMAT function might put those assigned female at birth at greater risk for sustained, pathological fear generalization over months or years (Cooper et al., 2022; Zoladz et al., 2022). Future evaluations of how functional PMAT polymorphisms are associated with (patho)psychological measures would be informative, as would inclusion of *SLC29A4* polymorphisms in pharmacogenomic evaluations for neuropsychiatric disorder risk and/or psychoactive drug treatment responses.

## Data Availability

The datasets presented in this study can be found in online repositories. The names of the repository/repositories and accession number(s) can be found at: https://osf.io/zea8r/?view_only=b93c68f010c442abb14a7162b6a79772.
