# Supplementary material for: PMAT enhances sexual dimorphism of fear behaviors and facilitates female mice’s generalized contextual fear extinction
Source: Front Neurosci. 2026 May 7;20:1693593. doi: 10.3389/fnins.2026.1693593 (PMC13189930; doi:10.3389/fnins.2026.1693593)
Supplement: Supplementary file 1 [file Data_Sheet_1.pdf]

Supplementary Material

1 Supplementary Materials and Methods

1.1 Animals

All experiments used adult (≥90 days old) male and female PMAT-deficient mice on a C57BL/6J background bred in-house. These mice were developed at the University of Washington by Dr. Joanne Wang[1] and are bred and used under a material transfer agreement between Kent State University and the University of Washington. Mice were group housed (2-5 per cage) within the same sex in cages containing 7090 Teklad Sani-chip bedding (Envigo, East Millstone, NJ, USA). All mice had *ad libitum* access to water and LabDiet 5001 rodent laboratory chow (LabDiet, Brentwood, MO, USA). Mouse housing rooms were maintained on a 12:12 light:dark cycle, with lights on at 07:00 local standard time. Room temperature was maintained at 22 ± 1°C and 40 ± 10% relative humidity. Male and female mice were run in experiments separately, with males always run before females. This was done to minimize males' behavior from being confounded by the presence of females' pheromones[2–4]. Kent State's Institutional Animal Care and Use committee approved all experiments, and conditions adhered to the National Research Council's Guide for the Care and Use of Laboratory Animals, 8th Ed.[5]. Power analyses for averaged context and cued comparisons (each involving 2 measurements) were performed *a priori* (repeated measures, within-between interactions; $f=0.025$; $\alpha=0.05$; $1-\beta=0.80$; corr=0.5; $\varepsilon=1$; G*Power v 3.1.9.6[6]), and indicated n=12 per sex and genotype (i.e., four groups).

1.2 Context fear conditioning

Mice underwent context fear conditioning[7–10] in Actimetrics chambers (internally, 18.7cm W x 20.6cm D x 20.1cm H; externally, 21.3cm W x 22.9 cm D x 25.7 cm H; Lafayette, IN) composed of two clear acrylic walls opposite each other, and joined by two aluminum walls. The training context (Context A), used both for context fear training and for testing context fear expression, consisted of a black and white striped background, shock grid floor, visible house light, and specific cleaning solution scent cue (70% ethanol). The novel context (Context B), used for testing context fear generalization, consisted of a black background, smooth floor, infrared light, and a different cleaning solution scent cue (Windex®). Freezing, i.e., absence of all movement except breathing, was quantified in real time using FreezeFrame 6 software (Actimetrics, Lafayette, IN). FreezeFrame 6 was also used to administer shocks during context training.

1.2.1 Context fear training

All mice underwent context fear training on day 0. Training involved five, 1s, pseudo-randomly administered scrambled foot shocks at 0.4 mA. Training lasted 6 min, with foot shocks administered at 137, 186, 229, 285, and 324 s. Data were exported in 5 s bins; post-shock freezing was calculated as average percent freezing over 30 s (i.e., six, 5 s bins), starting immediately after the 5 s bin during which each foot shock occurred.

1.2.2 Context fear expression, generalization, and discrimination testing

Mice were tested four weeks after training (day 28) then again 48 h thereafter (day 30). Context fear expression was tested in the training context (A), whereas context fear generalization was tested in the novel context (B). Testing in these two contexts was counterbalanced, meaning mice were either tested for fear expression at day 28, followed on day 30 by testing for fear generalization (A→B); or mice were tested for fear generalization at day 28, followed on day 30 by fear expression testing (B→A). Within sex and genotype, assignment to testing context order was systematically randomized to ensure acquisition confounds were unlikely to affect testing outcomes. Context testing lasted 10 min each day, and no shocks were administered on any testing day. Freezing was recorded in 30 s bins for the duration of each 10 min test. These testing data were analyzed both as a time course across all 10 min, and as the average percent freezing during minutes two through six across those respective ten, 30s bins[7–9].

1.3 Cued fear conditioning

Cued fear conditioning[8,11] was performed in the same Actimetrics chambers as for context fear conditioning. Cued fear training likewise used the same Context A as described for context fear conditioning. Testing of cued fear occurred in a novel context, similar to that described for context fear conditioning, save that floors were stainless steel with circular holes. Computer audio output volume was always set at 50% in Windows 11 Home (Microsoft; Redmond, WA). FreezeFrame 6 software was used to administer foot shocks and all auditory tones, as well as to measure freezing behavior. Two auditory cues were utilized: one tone at 7.5 kHz (paired conditioned stimulus, CS+), and one tone at 2.0 kHz (unpaired conditioned stimulus, CS−). Auditory cues were always played for 30s, and CS+ and CS− tones never coincided. The amplitude within FreezeFrame 6 for CS+ tones was set at 80, while for CS− tones it was set at 14. Before training occurred, decibels in each chamber were measured and ranged between 66-74dB for both tones to ensure comparable tone amplitude.

1.3.1 Cued fear training

All mice underwent training on day 0 in the training context. During training, mice were pseudo-randomly presented with five CS+ and five CS− tones. CS+ tones were played at 130, 300, 500, 710, and 910s, and each of these were paired with a 1s co-terminating shock at 0.4 mA. CS− tones were played during 210, 410, 650, 820, and 1000s, and were never paired with any aversive stimulus. Freezing was analyzed as the percent freezing during the 30s tones presented for each CS+ and CS−.

1.3.2 Cued fear expression testing, extinction training, and extinction retention testing

Mice experienced expression testing and extinction training on day 2, followed by extinction retention testing on day 4. No shocks were administered during any cued fear testing. Testing occurred in the same novel context as described for context fear testing, except the floor was stainless steel with circular holes with 0.635cm diameters. During day 2 (cued fear expression testing and extinction training), a total of 30 CS+ and 5 CS− tones were played over 37 min. CS+ tones were played during 120, 180, 240, 300, 360, 480, 540, 600, 660, 720, 840, 900, 960, 1020, 1080, 1200, 1260, 1320, 1380, 1440, 1560, 1620, 1680, 1740, 1800, 1920, 1980, 2040, 2100, and 2160s. CS−

tones were played at 420, 780, 1140, 1500, and 1860s[11]. For day 4 (extinction retention testing), 15 CS+ and 5 CS− tones were presented across 22 min. CS+ tones were played at 120, 180, 240, 300, 360, 480, 540, 600, 660, 720, 840, 900, 960, 1020, and 1080s. Similarly, CS− tones were played at 420, 780, 1140, 1500, and 1860s. Freezing during each day's five CS− tones was recorded and graphed individually, or averaged to create a single CS− freezing value each for days 2 and 4. Freezing during the 30 (day 2) or 15 (day 4) CS+ tones presented were averaged in sequential groups of three to evaluate time course of freezing across 10 (day 2) or 5 (day 4) points. For comparison with the single averaged CS− freezing value per mouse, described above, freezing for the first five CS+ tones were averaged per mouse[11].

1.4 Generalization and discrimination evaluations across fear paradigms

Discrimination and generalization are two ends of a spectrum[12,13], and consequently no universal thresholds exist at which one ends and the other begins. Rather, these processes are identified by utilizing previously established discriminable contexts or cues, and comparing how the procedurally optimized discrimination or generalization in the control group (here, +/+ mice) compares to that of the experimental manipulation group (here, +/− mice), as well as to how mice within each group respond to each of the contexts or cues they encountered during testing. For instance, testing context fear in Context B 28 days after training is an established timeline for producing context fear generalization in rodents[7,10,14–16]. If +/− mice displayed increased or decreased levels of fear in Context B on day 28 relative to +/+ mice, this would be one way to respectively detect enhanced context fear generalization or discrimination. Likewise, elevated or attenuated freezing of +/− mice during CS− testing, versus +/+ mice, respectively would suggest augmented cued fear generalization or discrimination[11,17].

1.5 Genotyping

Mice were weaned at postnatal day 21, at which time ear punches were taken for identification and genotyping. Genomic DNA was extracted from ear punches by digesting the 2 mm punches with Proteinase K (Roche, Basel, Switzerland) dissolved to 0.077% w/v the day of extraction with STE buffer (100mM Tris, 0.2% SDS, 200mM NaCl, and 5mM EDTA, pH=8.5)[1,18,19]. After a 2 h incubation at 55°C, DNA from the resulting supernate was precipitated with isopropanol, then pelleted DNA was reconstituted in TE buffer (10 mM Tris, 0.1 mM EDTA, pH=8). DNA amplification reactions occurred in 1X PCR buffer containing 1.74mM $MgCl_2$ and 34.7 mM dNTPs, 0.39 mM of each primer, and 0.20 mL of Platinum Taq (Invitrogen, Carlsbad, Ca, USA) per 25.6 mL reaction. Sequences of primers, designed by Duan and Wang[1], have been published previously[1,18,19]. Cycling conditions: 95 °C for 5 min; 34 cycles of 94 °C for 30 s, 59 °C for 30 s, and 72 °C for 90 s; 72 °C for 5 min; hold at 4 °C. Products were visualized using a 1% agarose gel electrophoresis ran at 150 V for 30 min. DNA amplicon sizes were referenced using a 1 kb DNA ladder (Invitrogen), with knockout alleles at 447 bp and wildtype alleles at 847 bp[1,18,19]. All genotypes were re-verified following experiment completion.

1.6 Statistical analyses

Analyses were performed with IBM SPSS Statistics (v 31.0.0.0 (117), IBM, Armonk, NY, USA). The threshold for significance was set *a priori* at $p<0.05$. Trends ($p<0.10$) that did not reach this threshold were only acknowledged in text if the associated partial η^2 (η_p^2) was ≥ 0.060. Cued data

were analyzed over time for each conditioning stage: 1) training; 2) fear expression testing and extinction training; 3) extinction retention testing. Context data were likewise analyzed over time at each stage: 1) training; 2) first testing; 3) second testing. These time course analyses were performed using two-way repeated measures (RM) general linear models (GLMs; time × sex × genotype), with Greenhouse-Geisser corrections for within-subjects analyses. Stage 2 cued averages of the first five CS+ and five CS− tones[11] were also analyzed with two-way RM GLMs (CS type × sex × genotype). Similarly, averages of context fear expression from minutes two through six of each testing day[7–9] were analyzed using two-way RM GLMs within context testing sequence (test day × sex × genotype). For all data sets, pairwise comparisons were also employed, using Bonferroni correction. One mouse (female +/−) in the cued experiment was excluded from all analyses due to failure to exceed 25% freezing for the first five CS+ tones during stage 2. One mouse (female +/+) in the context experiment was similarly excluded after failure to exceed 25% freezing during any training post-shock period. Two mice (one female +/+, one male +/−) tested for fear expression at day 28 were excluded because they failed to achieve ≥50% freezing for at least one 30 s bin. Finally, one mouse (female +/+) was excluded as an outlier, with day 28 fear expression average freezing more than 7 standard deviations below the group mean. GraphPad Prism (v 10.6.1 (799), GraphPad Software, San Diego, CA, USA) was used to generate figures showing estimated marginal means (EM means) ± 95% confidence intervals (CIs) calculated in SPSS.

2 References

1. Duan H, Wang J. Impaired Monoamine and Organic Cation Uptake in Choroid Plexus in Mice with Targeted Disruption of the Plasma Membrane Monoamine Transporter (Slc29a4) Gene. *J Biol Chem*. 2013;288(5):3535-3544. doi:10.1074/jbc.m112.436972
2. Baum MJ, Keverne EB. Sex Difference in Attraction Thresholds for Volatile Odors from Male and Estrous Female Mouse Urine. *Horm Behav*. 2002;41(2):213-219. doi:10.1006/hbeh.2001.1749
3. Kavaliers M, Choleris E, Colwell DD. Brief Exposure to Female Odors "Emboldens" Male Mice by Reducing Predator-Induced Behavioral and Hormonal Responses. *Horm Behav*. 2001;40(4):497-509. doi:10.1006/hbeh.2001.1714
4. Musso AE, Gries R, Zhai H, Takács S, Gries G. Effect of Male House Mouse Pheromone Components on Behavioral Responses of Mice in Laboratory and Field Experiments. *J Chem Ecol*. 2017;43(3):215-224. doi:10.1007/s10886-017-0819-y
5. National Research Council. *Guide for the Care and Use of Laboratory Animals*. 8th ed. National Academies Press (US); 2011. doi:10.17226/12910
6. Faul F, Erdfelder E, Lang AG, Buchner A. G*Power 3: A flexible statistical power analysis program for the social, behavioral, and biomedical sciences. *Behav Res Methods*. 2007;39(2):175-191. doi:10.3758/bf03193146
7. Beaver JN, Weber BL, Ford MT, et al. Generalization of contextual fear is sex-specifically affected by high salt intake. *PLOS ONE*. 2023;18(7):e0286221. doi:10.1371/journal.pone.0286221
8. Weber BL, Nicodemus MM, Hite AK, et al. Heterotypic Stressors Unmask Behavioral Influences of PMAT Deficiency in Mice. *Int J Mol Sci*. 2023;24(22):16494. doi:10.3390/ijms242216494

9. Lynch JF, Winiecki P, Gilman TL, Adkins JM, Jasnow AM. Hippocampal GABAB(1a) Receptors Constrain Generalized Contextual Fear. *Neuropsychopharmacol*. 2017;42(4):914-924. doi:10.1038/npp.2016.255

10. Cullen PK, Gilman TL, Winiecki P, Riccio DC, Jasnow AM. Activity of the anterior cingulate cortex and ventral hippocampus underlie increases in contextual fear generalization. *Neurobiol Learn Mem*. 2015;124:19-27. doi:10.1016/j.nlm.2015.07.001

11. Gilman TL, DaMert JP, Meduri JD, Jasnow AM. Grin1 deletion in CRF neurons sex-dependently enhances fear, sociability, and social stress responsivity. *Psychoneuroendocrino*. 2015;58:33-45. doi:10.1016/j.psyneuen.2015.04.010

12. Struyf D, Zaman J, Vervliet B, Diest IV. Perceptual discrimination in fear generalization: Mechanistic and clinical implications. *Neurosci Biobehav Rev*. 2015;59:201-207. doi:10.1016/j.neubiorev.2015.11.004

13. Bergstrom HC. Assaying Fear Memory Discrimination and Generalization: Methods and Concepts. *Curr Protoc Neurosci*. 2020;91(1):e89. doi:10.1002/cpns.89

14. Poulos AM, Mehta N, Lu B, et al. Conditioning- and time-dependent increases in context fear and generalization. *Learn Memory*. 2016;23(7):379-385. doi:10.1101/lm.041400.115

15. Wiltgen BJ, Silva AJ. Memory for context becomes less specific with time. *Learn Mem*. 2007;14(4):313-317. doi:10.1101/lm.430907

16. Lotz FN, Guerra KTK, Crestani AP, Quillfeldt JA. Multiple context discrimination in adult rats: sex variability and dynamics of time-dependent generalization of an aversive memory. *Learn Mem*. 2025;32(5-6):a054081. doi:10.1101/lm.054081.124

17. Pollack GA, Bezek JL, Lee SH, Scarlata MJ, Weingast LT, Bergstrom HC. Cued fear memory generalization increases over time. *Learn Mem*. 2018;25(7):298-308. doi:10.1101/lm.047555.118

18. Gilman TL, George CM, Vitela M, et al. Constitutive plasma membrane monoamine transporter (PMAT, Slc29a4) deficiency subtly affects anxiety-like and coping behaviours. *Eur J Neurosci*. 2018;48(1):1706-1716. doi:10.1111/ejn.13968

19. Beaver JN, Weber BL, Ford MT, Anello AE, Kassis SK, Gilman TL. Uncovering Functional Contributions of PMAT (Slc29a4) to Monoamine Clearance Using Pharmacobehavioral Tools. *Cells*. 2022;11(12):1874. doi:10.3390/cells11121874
